# Atypical Guillain-Barré Syndrome in a Pediatric Patient With a Preceding Non-COVID-19 Coronavirus Infection: A Case Report

**DOI:** 10.7759/cureus.59068

**Published:** 2024-04-26

**Authors:** Navjot Azad, Ajay Mittal, Michael Marzullo

**Affiliations:** 1 Integrative Medicine, Franciscan Heart and Vascular Associates, Monroe, USA; 2 Internal Medicine, Edward Via College of Osteopathic Medicine, Monroe, USA; 3 Nephrology, University of Florida College of Medicine, Ocala, USA; 4 Pediatrics, Christus St. Frances Cabrini Hospital, Alexandria, USA

**Keywords:** guillain-barré syndrome, pediatric critical care usa, pediatrics immunology, ophthalmology, pediatrics emergency

## Abstract

This study examines a four-year-and-one-month-old male with no significant past medical, family, or surgical history who initially presented to the pediatric clinic with cough, rhinorrhea, conjunctivitis, emesis, leg and arm pain, and increased difficulty ambulating. The patient was transferred to the emergency department and tested positive for a non-COVID-19 coronavirus infection. The patient was stabilized, given intravenous fluids, and discharged only to return to the clinic the next day with the onset of a headache, right eye ptosis, an inability to bear weight, and bilateral upper and lower extremity weakness resulting in an ataxic gait. In addition to the neurological deficits, the patient was found to have an elevated blood pressure and pulse. The patient was promptly transferred to a tertiary care clinic. Through exclusion of various differentials via testing, the patient was diagnosed and managed for atypical Guillain-Barré syndrome. Targeted therapies were initiated to prevent dysautonomia-associated morbidity. Following management, the patient’s condition vastly improved and he was admitted to rehabilitation bringing him back to optimal health. This study underlines the importance of prompt identification of atypical presentations of Guillain-Barré syndrome which may aid in avoiding preventable morbidity and mortality.

## Introduction

Guillain-Barré syndrome, the leading cause of flaccid paralysis in the United States, is associated with various gastrointestinal and respiratory pathogens, with the most common being *Campylobacter jejuni *followed by Cytomegalovirus, and Epstein-Barr virus [1-3]. In fact, 70% of patients with Guillain-Barré syndrome report an antecedent illness one to six weeks before their clinical presentation [4]. Furthermore, clinicians must be cognizant of respiratory failure as it is the most common cause of mortality in Guillain-Barré syndrome, occurring in up to 30% of patients [2]. To avoid significant morbidity and mortality, identification and management of Guillain-Barré and its atypical variants is of utmost importance.

Molecular mimicry plays a substantial role in the understanding of Guillain-Barré’s pathophysiology. Infectious agents invade the human host and present membranous components similar to the gangliosides present on the peripheral nerves of humans [5]. Exposure to these components leads to the production of anti-ganglioside antibodies resulting in an autoimmune response [5]. Common antibodies include anti-GD1a, anti-GM1, and anti-GQ1B. These antibodies have various different peripheral nerve targets which ultimately play a role in the heterogeneity of the clinical presentation of Guillain-Barré [6,7]. A typical presentation of Guillain-Barré is that of symmetric ascending neuromuscular weakness and loss of sensation [4]. Areflexia and hyporeflexia are also commonly seen. Electromyography and nerve conduction studies can be helpful in the diagnosis and often reveal slowed nerve conduction velocities with prolonged or absent F waves and H-reflex latencies [8,9]. Lumbar puncture cerebrospinal fluid analysis shows a classic pattern of albuminocytological dissociation in which there is a normal white blood cell count and an elevated cerebrospinal fluid protein count [10,11].

## Case presentation

A four-year-and-one-month-old Caucasian male presented to the pediatric clinic with cough, rhinorrhea, and conjunctivitis. The patient also reported one episode of emesis with arm and leg pain and the inability to ambulate warranting evaluation in the emergency department where the patient tested positive for a non-COVID-19 coronavirus infection. The patient had no family history of autoimmune conditions, had no past surgical history, and was born at term without any complications. Patient only reported an allergy to amoxicillin. He was estimated to be 93rd percentile when it came to weight-for-age and 39th percentile for height-for-age. The patient was managed supportively with intravenous normal saline followed by oral rehydration therapy, low-flow oxygen via nasal cannula at a flow rate of 1.5 L/kg/min, and once stabilized, was discharged.

The patient returned to the pediatric clinic the next day due to the persistence of symptoms. Assessment in the clinic revealed headache, vomiting, photophobia, right eye ptosis, and severe lethargy. Patient was unable to bear any weight and revealed bilateral upper and lower extremity weakness resulting in an abnormal gait. The clinic proceeded to transfer the patient to a tertiary care facility for an acute neurologic deficit of uncertain etiology.

Inpatient management included a neurology consult for bilateral upper and lower extremity weakness with an ataxic gait, an ophthalmology consult for right eye ptosis and photophobia, and a nephrology consult due to abnormal vitals revealing elevated blood pressure and pulse. In addition to appropriate consultations, the patient was managed with 15 mg/kg/dose PO acetaminophen q.i.d, 5 mg/kg/dose PO gabapentin t.i.d, and 0.5 mg/kg/dose PO morphine q.i.d for pain, 2 mg PO amlodipine q.d., and 1-2 mg PO isradipine prn for elevated blood pressure, intravenous dextrose and sodium chloride for fluid and electrolyte replenishment, intravenous immunoglobulin 2 g/kg/48 h, and nozin nasal sanitizer nasal liquid to reduce the risk of nasal pathogen transmission.

Nephrology was consulted and a 24-hour vital assessment revealed a max blood pressure of 150/93 and max pulse of 150 bpm, concerning dysautonomia. Urinalysis revealed 3+ ketones and was positive for nitrites revealing an underlying urinary tract infection managed with trimethoprim/sulfamethoxazole. Assessment of the heart was performed with an echocardiogram revealing normal left ventricular and right ventricular systolic function and an absence of pericardial effusion. Sonography revealed normal kidneys with a normal duplex Doppler evaluation of renal vasculature without evidence of renal artery stenosis. Abdominal ultrasound was also unremarkable. Nephrology plan included beginning the patient on 2 mg PO amlodipine q.d. for a target blood pressure <95th percentile for age (<111/69) and increasing the dose of PO isradipine from 1 mg to 2 mg if needed for additional blood pressure control.

Ophthalmology was consulted and confirmed photophobia and right eye ptosis. Extraocular movement examination revealed a right ophthalmoplegia adduction disorder. The patient denied blurry vision or double vision. Differential now included viral meningitis or encephalitis or possible myasthenia gravis requiring serum anti-acetylcholine receptor ab testing. Lumbar puncture cerebrospinal fluid analysis and blood testing did not confirm any of the differential diagnoses.

Neurology consultation revealed complete blood count, complete metabolic profile, and erythrocyte sedimentation rate/C-reactive protein/creatine kinase within normal limits. Bacterial and viral panels were also negative. A lumbar puncture was performed with subsequent cerebrospinal fluid analysis (Table 1). With a normal white blood cell count and elevated protein count, findings revealed albuminocytological dissociation, typical for Guillain-Barré syndrome. Magnetic resonance imaging brain, magnetic resonance imaging total spine, magnetic resonance venography, and magnetic resonance angiography with and without contrast were normal. Although electroencephalogram findings revealed minor slowing diffusely, this could be attributed to the sedative medications the patient was on. IgG index, serum and cerebrospinal fluid autoimmune panel, and ganglioside antibody testing revealed no abnormal findings.

**Table 1:** Cerebrospinal fluid (CSF) analysis.

CSF test parameters	Reference range	Observed value
Appearance	Clear	Clear
Opening pressure (mm H_2_O)	50-250	170
White blood cell count (x10^6^/L)	<5	3
Cell differential	Predominantly lymphocytes	Predominantly lymphocytes
Glucose (mg/dL)	50-80	76
Protein (g/L)	0.2-0.4	0.68

Sensory examination revealed intact light touch in all extremities. Deep tendon reflex testing revealed 2+ reflexes in the biceps, brachioradialis, knee, and ankle reflexes bilaterally. Coordination testing revealed very mild dysmetria and a tremor bilaterally. While the typical presentation of Guillain-Barré syndrome is that of ascending paralysis, loss of sensation, and loss or absence of deep tendon reflexes, the finding of albuminocytological dissociation raised suspicion for an atypical Guillain-Barré syndrome presenting with right eye ophthalmoplegia, right eye ptosis, and significant autonomic instability with tachycardia and hypertension. Neurology plan included beginning the patient on intravenous immunoglobulin and continuing monitoring of medication-controlled dysautonomia and respiratory status.

The patient was given intravenous immunoglobulin 2 g/kg/48 h over a four-day period and showed a significant improvement in symptoms. The patient was admitted to acute rehabilitation for an intensive and comprehensive program involving physical, speech, music, and recreational therapy, neuropsychology consult, and family training for 3 hours a day for five days a week. Rehabilitation was aimed at addressing any functional deficits sustained secondary to atypical Guillain-Barré syndrome. The patient was to continue on gabapentin and acetaminophen as well as amlodipine and isradipine for autonomic dysfunction. With the completion of inpatient management and rehabilitation, the patient showed significant improvement in static and dynamic sitting and standing, balance, fine motor skills, pain, and dysautonomia symptoms. The discharge plan included nephrology follow-up for blood pressure management and neurology follow-up for the management of dysautonomia and weakness.

## Discussion

Guillain-Barré syndrome (GBS) has more recently been of concern to clinicians due to its heterogeneous array of presentations. The most common being acute inflammatory demyelinating polyradiculoneuropathy followed by acute motor axonal neuropathy, acute sensory and motor axonal neuropathy, and the Miller-Fisher variants (characterized by ophthalmoplegia, ataxia, and areflexia) [12]. Still, over time, we are realizing just how widely diverse the presentation of Guillain-Barré truly is.

A study published in 2012 by the Iranian Journal of Child Neurology, examining 33 pediatric patients with GBS, reported that the most common preceding infections were respiratory (14 cases, 42.4%) followed by gastrointestinal (11 cases, 33.3%) [13]. Additionally, a literature review of GBS associated with preceding coronavirus in two pediatric patients reported the time from onset of coronavirus symptoms to clinical signs of GBS ranging from one to six weeks. In our report, we present a similar pediatric patient with preceding coronavirus-associated symptoms followed promptly by neurologic and autonomic deficits associated with GBS.

Another study published by Nasiri et al. evaluated the clinical features of 57 pediatric patients between the ages of one and 13 years with GBS and found the most common clinical presentations to be distal lower limb weakness (53 cases, 92.11%), reduced deep tendon reflex (DTR) (47 cases, 82.46%), and neuropathic pain (43 cases, 75.44%) [14]. These findings are parallel with those observed in the present study.

The present case presents just one more variant with the atypical constellation of ophthalmoplegia, ptosis, and significant tachycardia and hypertension. While labs reveal the classic feature of albuminocytological dissociation, there is an absence of typical motor, sensory, and nerve conduction/electromyography findings. The challenge introduced by a large number of variants is being able to exclude similarly presenting differentials and to promptly and precisely diagnose Guillain-Barré in order to prevent major morbidity and mortality brought upon by respiratory and cardiac distress. Once diagnosed, the management of all forms of Guillain-Barré follows a similar course with the hallmark being the administration of intravenous immunoglobulin while monitoring respiratory function and cardiac status in all patients [15,16]. Outpatient follow-up with rehabilitation for patients with incomplete recovery of motor and sensory function results in significant clinical improvement.

## Conclusions

Guillain-Barré is a complex autoimmune disorder with various etiologies including antecedent infections, medications, and surgeries. In addition to its various etiologies, Guillain-Barré has a heterogeneous presentation in widely varying populations. Challenges arise when the presentation strays from typical findings. Therefore, it is crucial for clinicians, without the presence of universal guidelines, to be able to diagnose and manage the disease promptly to help avoid morbidity and mortality. This study presented one such unique variant and adds to the growing body of work that may be useful to clinicians and healthcare workers.
